# Live Recombinant NDV-Vectored H5 Vaccine Protects Chickens and Domestic Ducks From Lethal Infection of the Highly Pathogenic H5N6 Avian Influenza Virus

**DOI:** 10.3389/fvets.2021.773715

**Published:** 2022-02-03

**Authors:** Jiho Lee, Deok-hwan Kim, Jinyong Noh, Sungsu Youk, Jei-hyun Jeong, Joong-bok Lee, Seung-Yong Park, In-soo Choi, Sang-Won Lee, Chang-seon Song

**Affiliations:** ^1^Department of Avian Disease, College of Veterinary Medicine, Konkuk University, Seoul, South Korea; ^2^KCAV Co., Ltd., Seoul, South Korea; ^3^Southeast Poultry Research Laboratory, Agricultural Research Service, U.S. Department of Agriculture, U.S. National Poultry Research Center, Athens, GA, United States

**Keywords:** highly pathogenic avian influenza, Newcastle disease virus, recombinant vaccine, vector vaccine, ducks, chickens

## Abstract

The H5 subtype highly pathogenic avian influenza virus (HPAIV) has been introduced to South Korea every 2 or 3 years *via* wild migratory waterfowls, causing devastating damages to the poultry industry. Although most damages and economic losses by HPAIV are focused on chicken layers, domestic ducks are known to play a major role in the farm-to-farm transmission. However, most HPAIV vaccine studies on poultry have been performed with oil-emulsion inactivated vaccines. In this study, we developed a live recombinant Newcastle disease virus (NDV)-vectored vaccine against H5 HPAIV (rK148/ES2-HA) using a previously established NDV vaccine strain (K148/08) isolated from a wild mallard duck. The efficacy of the vaccine when administered *via* the oculonasal route or as a spray was evaluated against lethal H5 HPAIV infection in domestic ducks and chickens. Oculonasal inoculation of the rK148/ES2-HA in chickens and ducks elicited antibody titers against HPAIV as early as 1 or 2 week after the single dose of vaccination, whereas spray vaccination in ducks elicited antibodies against HPAIV after the booster vaccination. The chickens and ducks vaccinated with rK148/ES2-HA showed high survival rates and low viral shedding after H5N6 HPAIV challenge. Collectively, vaccination with rK148/ES2-HA prevented lethal infection and decreased viral shedding in both chickens and ducks. The vaccine developed in this study could be useful in suppressing the viral shedding in H5 HPAIV outbreaks, with the ease of vaccine application and fast onset of immunity.

## Introduction

Influenza virus type A is an RNA virus belonging to the family *Orthomyxoviridae* and can infect a variety of host species, including humans, pigs, and birds. Wild birds are considered to be natural reservoirs of the virus, and poultry are highly susceptible to the virus. Therefore, wild birds and poultry are the major factors in the transmission of avian influenza (1, 2). Avian influenza virus can be classified depending on the antigenic diversity of the two surface glycoproteins, namely, hemagglutinin (HA) and neuraminidase (NA); there are 16 subtypes from H1 to H16, and 9 subtypes from N1 to N9 (2). The combination of these two proteins theoretically allows 144 subtypes to exist (3). Avian influenza virus is classified into the highly pathogenic avian influenza virus (HPAIV) and low pathogenic avian influenza virus (LPAIV) based on pathogenicity testing in chickens and molecular determination of the HA protein (2). To date, the majority of HPAIVs originate from the H5 or H7 subtypes (1).

Since the emergence of the A/goose/Guangdong/1996 (Gs/GD) lineage H5N1 HPAIV in China, H5 HA has been genetically subdivided into 10 clades, from clades 0 to 9 (4). The continuous spread and evolution of the clade 2 H5 subtype of AIV expanded this clade into clade 2.1 and clade 2.3.4 between 2006 and 2008, and further into clade 2.3.4.4. The clade 2.3.4.4 H5 viruses are transmitted to many countries *via* wild migratory waterfowls, causing serious economic damage to the poultry industry between 2016 and 2018 (5).

Although the Gs/GD lineage H5 HPAIV caused devastating damage in poultry, the mortality, and severity of the infection varied in duck species, ranging from asymptomatic to lethal infection (6–9). Because duck species are susceptible to HPAIV but show low pathogenicity, ducks can act as “Trojan horses” to spread HPAIV to other species (10). While asymptomatic wild migratory ducks contribute to the spread of HPAIV, domestic ducks act as a healthy reservoir epidemiologically (9, 11). Although high biosecurity and culling or stamping of infected birds are effective ways to control the spread of the influenza virus from wild birds to poultry, these measures are impractical to enforce and are difficult to apply when the viruses are actively circulating from one farm to another (11). Therefore, vaccination in ducks should be prepared for a contingency as part of a comprehensive control strategy with other biosafety measures. However, vaccination coverage is poor in domestic ducks because of the difficulty in vaccinating a sufficient number of ducks, especially with ducks reared in open fields, and few studies have been conducted on the vaccination of domestic ducks in the field (11, 12).

Most HPAIV vaccines used in domestic ducks are oil-emulsion-inactivated vaccines (11). However, inactivated vaccines require labor-intensive administration methods, and it is difficult to administer a vaccine to a sufficient number of ducks to maintain herd immunity (11). Live attenuated HPAIV vaccines are not recommended because of the risk of mutation and reassortment with other avian influenza viruses. Alternatively, live vector vaccines have advantages over inactivated and live attenuated vaccines; they are easy to administer by spraying or drinking water and less likely to cause reassortment and relapse of virulence. While a few viral species, including Newcastle disease virus (NDV), turkey herpesvirus, and Marek's disease virus have been used as a recombinant vector vaccine against avian influenza for use in gallinaceous poultry (13–15), only duck enteritis virus has been developed for use in domestic ducks (16, 17). However, no vector vaccines have been designed to encompass both gallinaceous poultry and domestic ducks.

NDV is a non-segmented, single-stranded, negative-sense RNA virus belonging to the family *Paramyxoviridae* under the genus *Avulavirus*. NDV encodes six structural proteins, namely nucleoprotein (NP), phosphoprotein (P), matrix (M), fusion (F), hemagglutinin-neuraminidase (HN), and large (L) proteins (18). NDV is categorized into lentogenic (avirulent), mesogenic (moderately virulent), and velogenic (virulent) based on the severity of the disease (19). The F protein of the mesogenic and velogenic strains has multiple basic amino acid sequences at the cleavage site. Proteolytic activity of ubiquitous subtilisin-like proteases, such as furin, causes systemic infection by virulent strains. By contrast, the lentogenic strain has an F protein cleavage site with fewer basic amino acid sequences and can only be cleaved by trypsin-like extracellular proteases that are confined to the respiratory and enteric tracts (20). Lentogenic strains have been widely used as live attenuated vaccines to control Newcastle disease in chickens (21).

The advent of the reverse genetic system allowed NDV to be used as a vector vaccine for animal and human diseases, especially against the influenza virus (22–25). Most studies on NDV vector vaccines against HPAIV have been performed on chickens. Currently, NDV vector vaccines against HPAIV are commercially available for poultry vaccination in China and Mexico (26–28). However, few studies have described the use of an NDV vector vaccine against HPAIV in ducks (29). Only one study, performed by Ferreira et al., demonstrated that a recombinant NDV vaccine expressing the HA protein of H5N1 elicited both humoral and cellular responses in mule ducks, with complete clinical and virological protection against the H5N1 HPAIV strain (30).

In our previous study, we isolated K148/08 NDV from the mallard ducks and demonstrated its safety, thermostability, immunogenicity, and protective efficacy against velogenic NDV (31). In the present study, we constructed the rK148/ES2-HA vaccine, a recombinant vector vaccine based on K148/08 NDV and expressing HA of clade 2.3.4.4e HPAIV H5N6 subtype virus (A/duck/Korea/ES2/16), the virus selected as an antigen candidate in the national AI antigen bank in South Korea (32). The protective efficacy of rK148/ES2-HA vaccine against H5N6 HPAIV in commercial ducks and specific-pathogen-free (SPF) chickens *via* oculonasal or spray vaccination was evaluated.

## Materials and Methods

### Cells and Viruses

Chicken embryo fibroblast (CEF) primary cells and Hep-2 (CCL-81; ATCC, Manassas, VA) cell lines were grown in Dulbecco's modified Eagle's medium supplemented with 8% fetal bovine serum and antibiotics. The previously established NDV vaccine (K148/08), KCTC 11570BP, was used to generate a vector vaccine platform (31). The modified vaccinia Ankara T7 recombinant virus (MVA-T7) for the supply of T7 reverse transcriptase was kindly provided by Dr. Bernard Moss. The MVA-T7 virus was propagated in CEF cells for further use. The H5 subtype HPAIV clade 2.3.4.4e strain H5N6 A/duck/Korea/ES2/16 (ES2), GISAID EpiFlu Isolate ID EPI_ISL_239262, was kindly provided by the Animal and Plant Quarantine Agency in Korea.

### RNA Preparation

The viral RNA of the K148/08 NDV strain was extracted using the RNeasy kit (Qiagen, Valencia, CA, USA). The propagation and RNA extraction of the ES2 HPAIV was performed inside the biosafety level 3 (BSL3) facility at Konkuk University, as described in sections Virus Rescue and Propagation and Serological Analysis. cDNAs of the K148 NDV full genome and ES2 HPAIV HA gene were synthesized using the SuperScript IV first-strand synthesis system (Thermo Fisher Scientific, California, USA).

### Construction of the Full-Length cDNA Clone of K148 Virus

**Figure 1:**
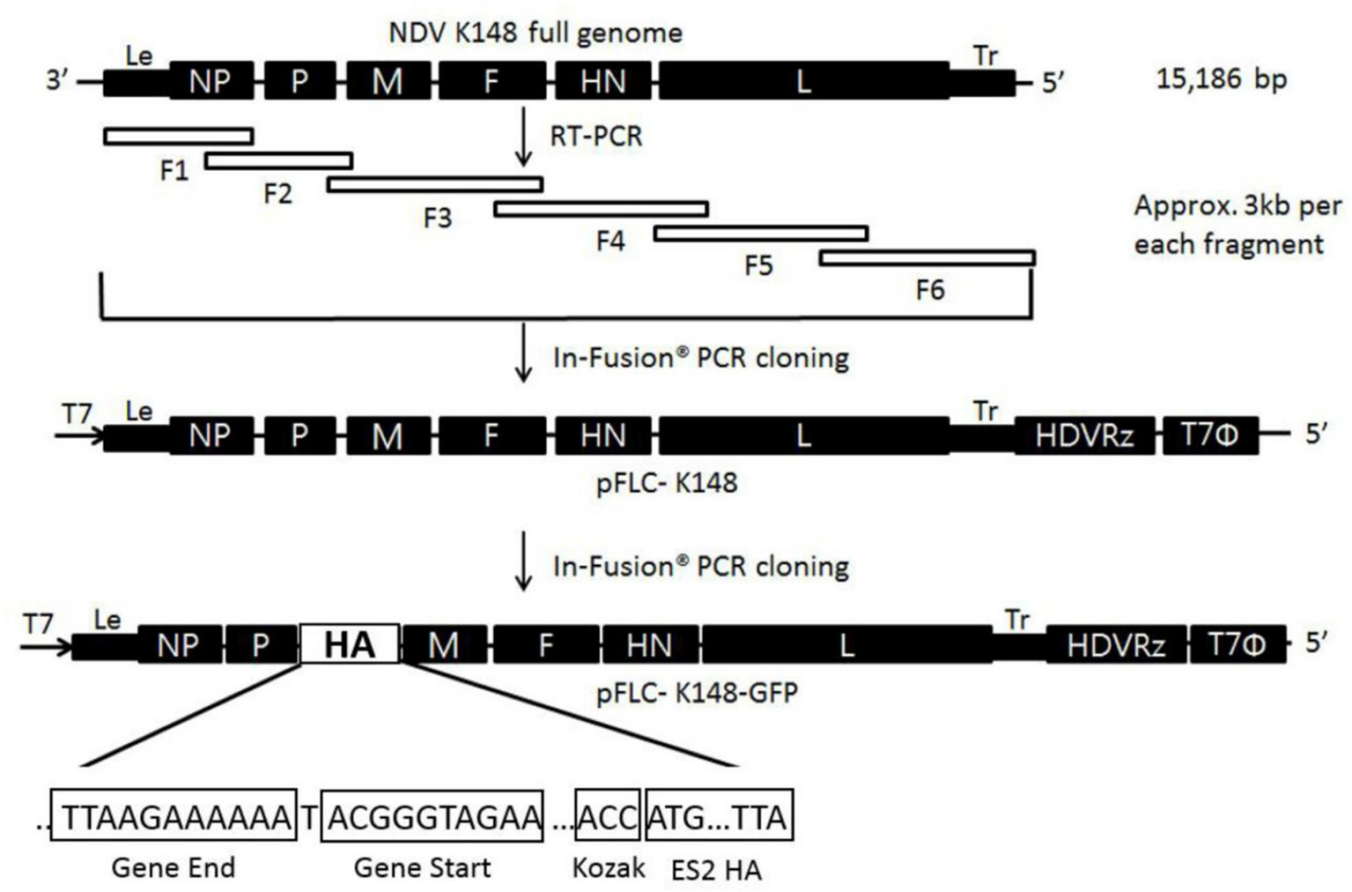
Schematic description of the construction of recombinant pK148/ES2. Six cDNA fragments of K148 were generated using RT-PCR. Each fragment was amplified and cloned into a TA vector and sequentially cloned into the pBlueScript vector using in-fusion PCR. The cDNA of the HA gene was generated and amplified as described above and inserted between the P and M genes of the linearized pK148 vector. HDV Rz represents the hepatitis delta virus ribozyme sequence and T7Φ represents the T7 terminator sequence.

The cDNA of K148 NDV was divided into six fragments with ~300 nucleotides of overlapping regions between adjacent fragments (Figure 1). All six fragments were amplified with six sets of gene-specific primers and cloned into the RBC TA cloning vector (RBC Bioscience, Taipei, Taiwan; Supplementary Table 1). Each fragment was sequentially inserted into the pBluescript vector using the In-Fusion® PCR cloning kit (Clontech, Mountain View, CA). The *pfu*Ultra^TM^ II fusion HS DNA polymerase (Stratagene, La Jolla, CA) was used to amplify the in-fusion fragments and linearizing vectors. The vector plasmid was linearized before each step of the fragment in-fusion. Nucleoprotein (NP), polymerase (P), and large polymerase (L) genes were cloned into an expression plasmid to construct the supporting plasmids. The primers used for vector linearization and in-fusion are listed in Supplementary Table 2. After construction of the full-length cDNA clone of the K148 virus (pK148), pK148 was transformed into HIT-DH5α competent cells (RBC Bioscience) at 37°C for 18 h and purified using the PureLink™ HiPure plasmid midiprep kit (Thermo Fisher Scientific).

### Construction of the K148 Clone Containing HA Gene of ES2 HPAIV

pK148 was linearized to insert the HA gene of ES2 HPAIV virus and an additional cDNA fragment containing the untranslated region (UTR), including the gene end, and gene start (GS) signals, between polymerase gene (P) and matrix (M) gene of the full genome K148 cDNA clone. The additional UTR and cDNA of ES2 HA gene were amplified by PCR and cloned into RBC TA cloning Vector (RBC Bioscience, Taipei, Taiwan). The multibasic cleavage site of the HA gene (PLRERRRKR/GLF) was replaced by the cleavage site of LPAIV (PLRERR/GLF). To enhance the expression of ES2 HA gene, Kozak sequence (ACC) was inserted at the upstream of HA gene by using Muta-direct^TM^ site-directed Mutagenesis kit (iNtRON Biotechnology, Korea). The cDNA of UTR and the HA gene were ligated and inserted between P and M genes of pK148 by infusion PCR described above, forming pK148/ES2-HA clone. The pK148/ES2-HA clone was amplified and purified as described above.

### Virus Rescue and Propagation

The plasmid with full-genome cDNA of pK148 or pK148/ES2-HA and three supporting plasmids were co-transfected into Hep-2 cells for virus rescue. Briefly, Hep-2 cells seeded on six-well-plates were infected with the MVA-T7 virus to provide T7 transcriptase. A mixture of 8.8 μg of pK148 or pK148/ES2-HA, 1 μg of pSupport_NP, 0.1 μg of pSupport_P, and 0.1 μg pSupport_L plasmids were transfected into Hep-2 cells using Lipofectamine 3000 (Thermo Fisher Scientific). After 1 h of transfection, the transfected cells were washed three times with phosphate-buffered saline and incubated in Opti-MEM at 37 °C for 72 h. After incubation, the cells and supernatants were frozen and thawed three times to release rK148 and rK148/ES2-HA viruse particles. The harvested viruses were inoculated into 10-day-old SPF embryonated chicken eggs and incubated at 37°C. After 3 days of incubation, the inoculated eggs were chilled at 4°C for 1 h and allantoic fluids were used for the hemagglutination assay to detect the rescued virus. HA-positive samples were filtered through a 0.2 μm syringe filter (Ministart RC 15, Sartorius Stedim Biotech, Germany) and subsequently passaged in 10-day-old SPF embryonated chicken eggs. Allantoic fluids were harvested, aliquoted, and stored at −80°C for further experiments. To determine the 50% egg infectious dose (EID_50_), rescued virus, or ES2 virus were 10-fold serially diluted and inoculated in 10-day of SPF embryonated chicken eggs. After 3 days of incubation, allantoic fluids were harvested and checked for hemagglutination reaction. The EID_50_ endpoint was calculated according to Reed and Muench method.

### Viral Purification and Western Blotting

To examine the expression of ES2 HA protein and the stability of the HA gene after consecutive passages, rK148 and rK148/ES2-HA passaged four times in 10-day-old SPF embryonated chicken eggs, were harvested and clarified using low centrifugation (2,000 × g, 10 min, 4°C). The supernatants of the clarified fluids were inactivated with 0.1% formalin. After inactivation, the supernatants were pelleted using centrifugation (30,000 × g, 1 h, 4°C). The pellets were resuspended in PBS, loaded on a 20–50% (w/v) discontinuous sucrose density gradient, and centrifuged (150,000 × g, 1.5 h, 4°C) for purification. Protein concentrations of the purified samples were measured using the Bradford protein assay (Pierce, USA). The purified samples with high concentration (2 μg/well) and low concentration (400 ng) were analyzed using Western blotting. The expression of the ES2 HA gene was detected using antiserum of chickens immunized with clade 2.3.4.4e H5 subtype AIV and HRP-conjugated goat anti-chicken IgY secondary antibody (Invitrogen, California, USA). The presence of NDV was identified with anti-NDV HN mouse IgG antibody, which was kindly provided by Bionote (Suwon, Republic of Korea), and HRP-conjugated goat anti-mouse IgG antibody (Komabio, Seoul, Republic of Korea).

### Viral Growth Properties

The rK148/ES2-HA virus and K148 NDV were inoculated in 10-day-old embryonated SPF chicken eggs with 0.1 mL of serially diluted virus (10^1^, 10^2^, 10^3^, and 10^4^ EID50). Each serial dilution of two viruses were inoculated in three 10-day-old embryonated SPF chicken eggs. The allantoic fluid from inoculated eggs were harvested at 12, 24, 36, 48, 60, and 72 h post inoculation and viral titers were measured in HAU (Hemagglutination Unit).

### Immuniation and Challenge Experiments

Twenty-four 1-day-old commercially available Pekin ducks were kindly provided by the Moran Food & Breeding Company (Eum-seong, Republic of Korea). Eighteen 1-day-old SPF chickens were purchased from Namduk SPF (Icheon, Republic of Korea). The ducks and chickens were divided into seven groups (*n* = 6), as described in Table 1. The vaccination groups were vaccinated oculonasally or by the spray method with a 10^7^ EID50 dose of rK148 or rK148/ES2-HA. An automated spraying device (SK-MO-2000, Threeshine Inc, Korea) producing aerosol particles of 50–70 μm, was used for the spray vaccination. The mock vaccination group was vaccinated with PBS. Booster vaccination was performed 3 weeks after the priming vaccination. The clinical signs of the vaccinated chickens and ducks were assessed daily. Blood samples were collected from ducks and chickens for serological analyses. Six weeks after priming, all birds were challenged with 100 mean bird lethal dose (BLD_50_) of ES2 HPAI virus. Each BLD_50_ for chickens or ducks was established as described previously (9, 33). The clinical signs and mortality rates were recorded daily. Oropharyngeal and cloacal swab samples of the challenged birds were collected at 2, 4, 6, 8, and 12 days post-challenge. All animal experiments conducted in this study were observed and approved by the Institutional Animal Care and Use Committee of Konkuk University (permit number: KU21045). The vaccination experiment was performed in a BSL2 facility, and the challenge study was performed at the animal BSL3 facility at Konkuk University.

**Table 1:** Vaccination and challenge schedule.

	**Vaccine**	**Number of animals**	**Vaccine route^a^**	**Live vaccine dose**	**Challenge dose**
Ducks	rK148	6	ON^b^	10^7^ EID_50_	100 BLD_50_
	rK148/ES2-HA	6	ON		(10^6.0^ EID_50_)
	rK148/ES2-HA	6	Spray		
	Mock (PBS)	6	ON		
Chickens	rK148	6	ON	10^7^ EID_50_	100 BLD_50_
	rK148/ES2-HA	6	ON		(10^5.8^ EID_50_)
	Mock (PBS)	6	ON		

a *Birds were inoculated when they were 1-day-old. Booster vaccination was administered at 3 weeks post-vaccination.*

b *ON, Oculonasal*.

### Serological Analysis

The antibody responses in chickens and ducks before and after vaccination with rK148/ES2-HA were determined using hemagglutination inhibition (HI) tests. As both the HA protein of AIV and HN protein of NDV expressed on rK148/ES2-HA can induce hemagglutination, inactivated K148 virus and ES2 HPAI virus were used as antigens. Briefly, sera collected before vaccination [0 weeks post-vaccination (wpv)] and at 1, 2, 3, 4, and 5 wpv were heat-inactivated (56°C for 30 min) to inactivate the complement system. Duck serum samples were treated overnight with receptor-destroying enzyme (Denka Seiken Co., Japan) at 37°C to eliminate non-specific HI factors. Inactivated serum was diluted two-fold in PBS and incubated with four hemagglutination units of K148/08 or ES2 antigen for 40 min. The incubated samples were then mixed with 1% chicken red blood cells in a 96-well V-bottom plate. The HI titer of each sample was determined following a standard protocol (2).

### Detection of Viral RNA Shedding After the Challenge

To quantify viral shedding after challenge, RNA was extracted from oral and cloacal swab samples collected on days 2, 4, 6, 8, and 12 post-challenge (pc). The RNA was extracted from 200 μL of the supernatant of swab samples using the Roche MagNa Pure 96 extraction system (Roche, Manheim, Germany) according to the manufacturer's instructions. The extracted RNA was quantified by analyzing the cyclic threshold (Ct) value using matrix gene-based real-time reverse transcription-polymerase chain reaction (rRT-PCR) (34). The Ct values were converted into an infectious unit equivalent to EID_50_/mL using a standard curve.

### Clinical Scoring

Chickens and ducks were monitored and scored every 24 h until 14 dpi. Chickens and ducks were scored based on their health status as follows: 0 (normal); 1 (sick); 2 (severely sick), and 3 (dead). “Sick” birds showed one of the clinical signs whereas “severely sick” ducks showed more than one: respiratory involvement, ruffled feathers, lethargy, anorexia, prostration, swollen heads, and green diarrhea.

### Statistical Analyses

The data for viral shedding titers and HI antibody titers were analyzed using one-way and two-way ANOVA with the Tukey–Kramer *post-hoc* test, and the long-rank test were performed to analyze significant of survival curves between animal experiment groups in GraphPad Prism version 8.0 (GraphPad Software Inc., CA). Statistical significance was set at *p* ≤ 0.05.

## Results

### Generation of rK148 and rK148/ES2-HA Viruses

**Figure 2:**
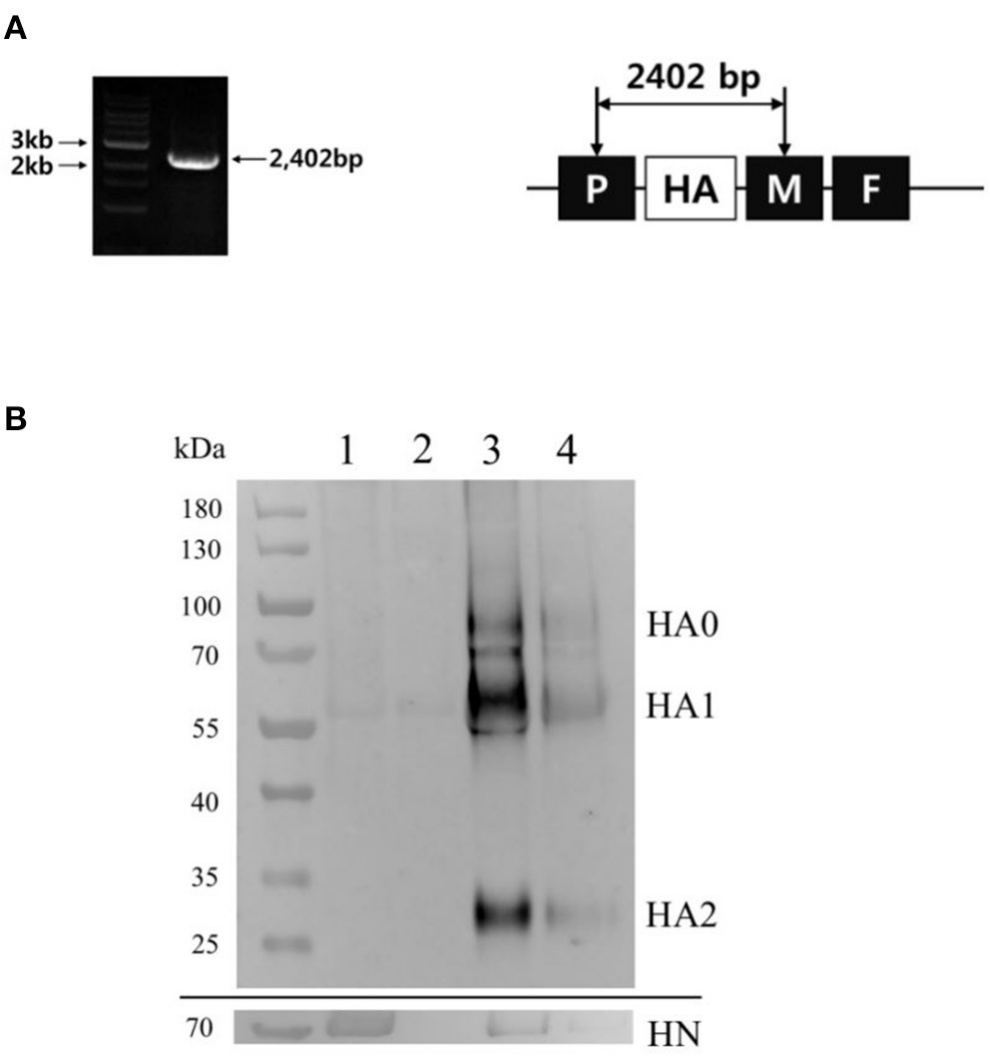
Identification of the genome insertion and the expression of ES2-HA protein by rK148/ES2-HA. **(A)** After the rescue and two sequential propagations in 9–11-day-old SPF chicken eggs, rK148/ES2-HA virus was harvested and checked for the insertion of the ES2-HA gene. The gene fragment, which includes the ES2 HA gene flanked by two gene-specific primers, was amplified and analyzed using gel electrophoresis. **(B)** The expression of ES2-HA protein was detected using Western blot analysis with chicken anti-clade 2.3.4.4e H5 AIV serum and HRP-conjugated goat anti-chicken IgY antibody. A monoclonal anti-NDV HN antibody was used to detect the HN protein of NDV with an approximate size of 70 kDa (lower panel). Lane 1: rK148 high concentration, lane 2: rK148 low concentration, lane 3: rK148/ES2-HA high concentration, lane 4: rK148/ES2-HA low concentration.

The pBlueScript encoding full genome of K148/08 virus cDNA (pK148) was generated using in-fusion PCR (Figure 1). The ES2-HA gene was inserted between the P and M junctions and ligated into the pK148 plasmid to construct pK148/ES2-HA (Figure 1). The rK148/ES2-HA virus was rescued after transfection of pK148/ES2-HA into Hep-2 cells. The rescue of rK148 and rK148/ES2-HA viruses was verified by using the hemagglutination assay, and the HA gene was verified by using PCR after three consecutive passages in 9–11-day-old embryonated SPF chicken eggs (Figure 2). No mutations were observed in the HA gene of the passaged rK148/ES2-HA virus. The total length of the gene encoding the K148/ES2-HA gene is 17,190 bp, which follows “the rule of six” (35).

### Expression of ES2-HA Protein by rK148/ES2-HA Virus

The expression of ES2-HA protein was evaluated using Western blot analysis after the concentration and purification of rK148/ES2-HA. The expression of ES2-HA protein in rK148/ES2-HA was demonstrated by detecting the ~70 kDa band representing the HA0 protein, the ~55 kDa band representing the HA1 protein, and the ~30 kDa band representing the HA2 protein. The HN proteins of rK148 and rK148/ES2-HA were detected in the ~70 kDa band (Figure 2B).

### Growth Properties of rK148/ES2-HA

**Figure 3:**
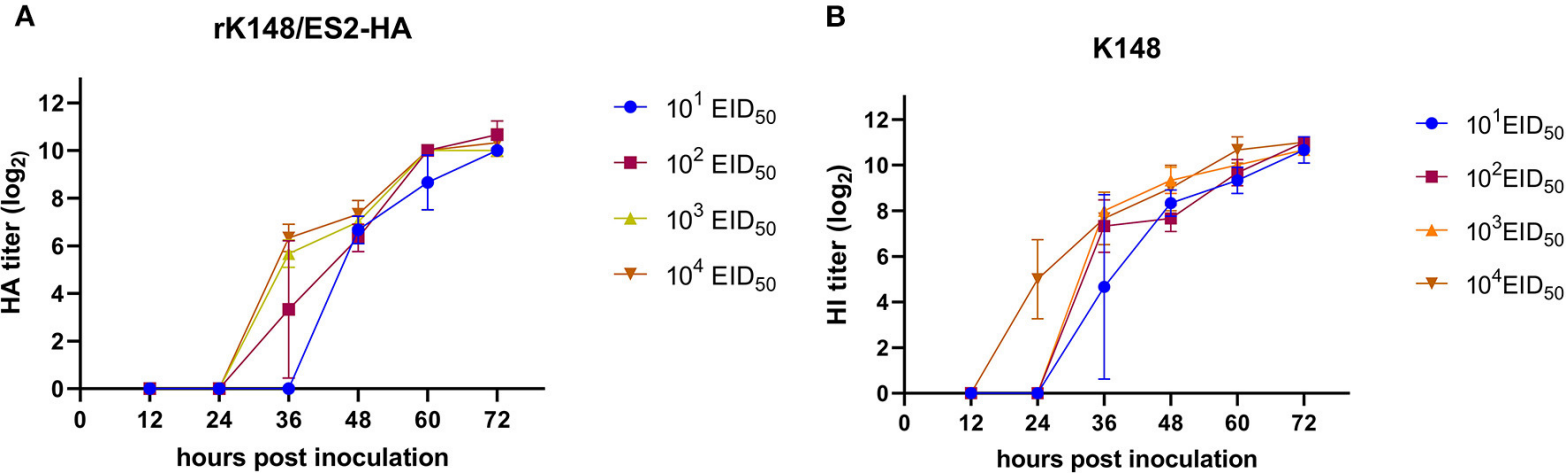
Viral growth kinetics of rK148/ES2-HA **(A)** and K148/08 **(B)** in 10-day-old SPF embryonated chicken eggs. Viral growth kinetics was performed by inoculating four serial doses (from 10^1^ to 10^4^ EID_50_) in 10-day-old SPF embryonated chicken eggs. Hemagglutination units were measured every 12 h after the inoculation.

The titer of rK148/ES2-HA (10^9.5^ EID_50_/mL) was slightly lower than that of the K148/08 backbone virus (10^9.7^ EID_50_/mL) after 72 h of incubation 10-day-old embryonated SPF chicken eggs. To compare the viral growth of rK148/ES2-HA and K148/08 NDV, four serial dilutions (10^1^-10^4^ EID50) were inoculated in 10-day-old embryonated SPF chicken eggs and the viral titers were measured in HAU every 12 h after the inoculation. The rK148/ES2-HA showed the highest mean HAU (2^10.67^) at 72 h of incubation, while the K148/08 showed the highest mean HAU (2^11^) at 72 h of incubation. No significant differences were observed between growth curves of rK148/ES2-HA and K148/08 NDV (Figure 3).

### The Immune Response of Vaccinated Animals Against HPAIV

**Figure 4:**
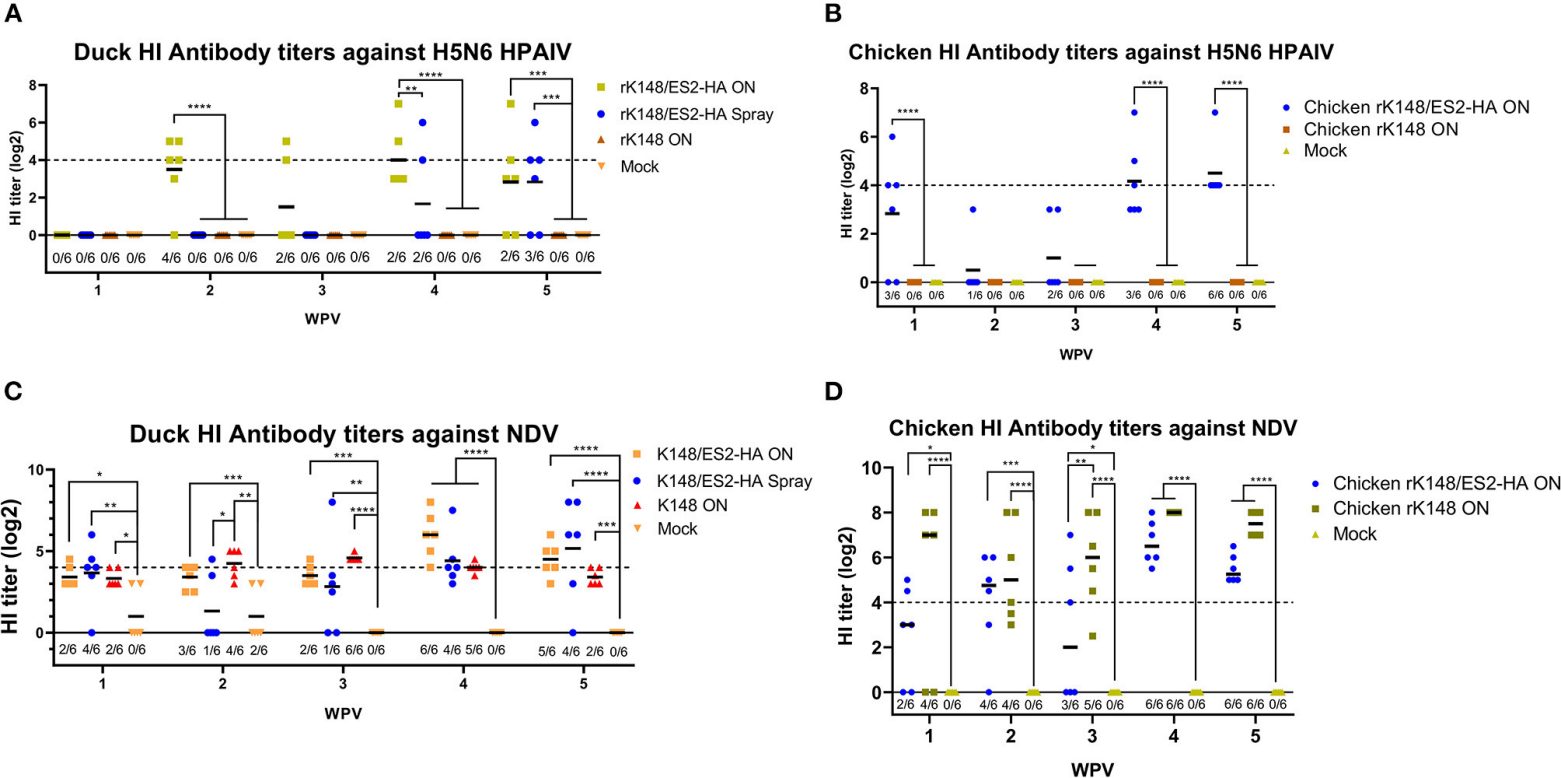
Hemagglutination inhibition assay of serum samples from vaccinated ducks and chickens. Six 1-day-old ducks from each group were vaccinated and booster-vaccinated at 3 wpv with 10^7^ EID50 of rK148/ES2-HA (ON), rK148/ES2-HA (Spray), K148 (ON), and PBS. Blood samples were collected every week after the immunization and used to measure hemagglutination inhibition (HI) antibody titer against clade 2.3.4.4e H5N6 HPAIV **(A,B)** and K148 NDV **(C,D)**. Ducks and chickens with HI antibody titers lower than 4 log_2_ were regarded as seronegative. (*p < 0.05, **p < 0.01, ***p < 0.001, and ****p < 0.0001 using ANOVA with *post-hoc* Tukey's test compared with other groups).

No antibodies against the ES2 virus were observed in the chickens and ducks before vaccination. The HI antibody titer against HPAIV of ducks immunized *via* the oculonasal route started to increase at 2 wpv with geometric mean titer (GMT) of 11.31. The HI titer of ducks immunized *via* the spray method increased 1 week after booster vaccination (GMT: 3.18), and ducks vaccinated *via* the oculonasal route showed a higher HI titer (GMT: 16) than the spray-vaccinated group (GMT: 3.18) at 4 wpv. The HI titer of the oculonasally vaccinated ducks decreased (GMT: 7.11), whereas the HI titer of the spray-vaccinated ducks increased (GMT: 7.11) at 5 wpv (Figure 4A). Since HI titer is regarded as positive if the inhibition at a serum dilution of 1/16 or more against 4 HAU of antigen, majority of ducks were HI negative against HPAIV after the vaccination, with only 2 and 3 ducks in the oculonasal and the spray group showed positive HI antibody titer against HPAIV at 5 wpv (Figure 4A). For chickens immunized with the rK148/ES2-HA vaccine, the HI titer to the homologous ES2 virus was elicited as early as at 1 wpv with a GMT of 7.1. After booster vaccination at 3 wpv, the HI titers increased with GMT of 17.96. The chickens vaccinated *via* the oculonasal route showed an increase in HI titer from 4 wpv (GMT:17.96) to 5 wpv (GMT: 22.63). Only at 5 wpv, all chickens in the rK148/ES2-HA group showed positive HI titer (≥4 log_2_) against ES2 HPAIV (Figure 4B). Chickens and ducks vaccinated with rK148 or PBS did not show HI antibody titers against H5N6 HPAIV (Figures 4A,B).

### Immune Response of the Vaccinated Animals Against NDV

Unlike HI antibody titer against HPAIV in ducks, low HI antibody titers against NDV were observed in all groups. The HI titer of the mock vaccinated group diminished after 3 weeks, while other vaccinated ducks showed slight increment in HI titer against NDV after the booster vaccination. The oculonasally vaccinated ducks showed the highest positive rate of 6/6 and GMT of 64 at 4 wpv, spray vaccinated ducks showed the highest positive rate of 5/6 and GMT of 35.93 at 5 wpv, and rK148 vaccinated ducks showed the highest positive rate of 6/6 and GMT of 23.97 at 3 wpv (Figure 4C). In the chicken experiment, preimmunized chickens and mock-vaccinated chickens did not show HI titers against NDV, whereas chickens immunized with rK148/ES2-HA and rK148 showed increases in HI titers after the priming and booster vaccinations (Figure 4D).

### Protection and Viral Shedding

**Figure 5:**
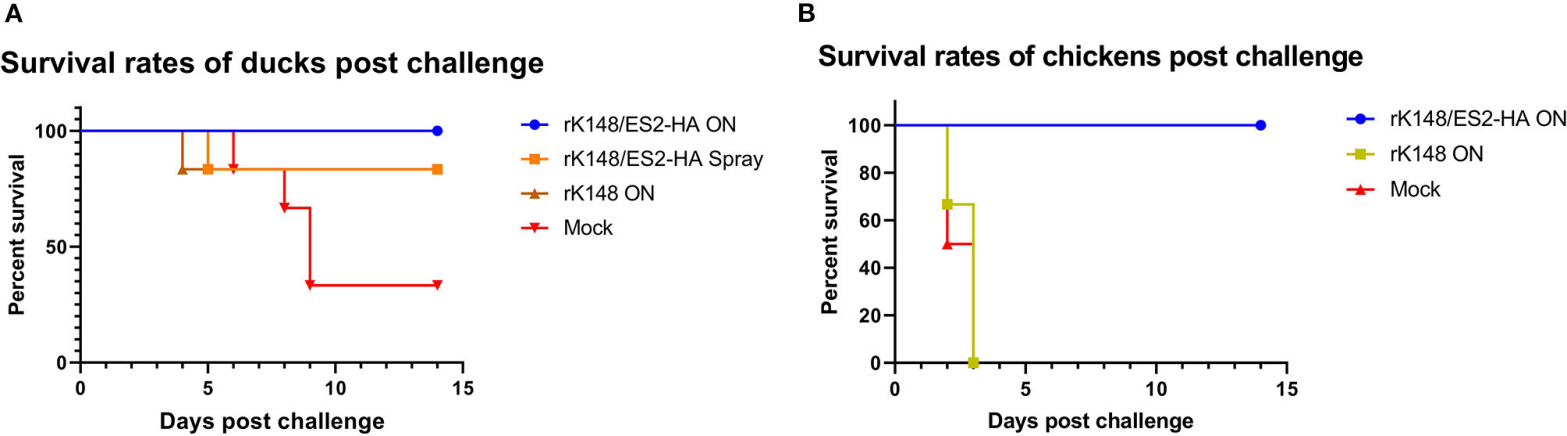
Survival rates of ducks **(A)** and chickens **(B)** after the challenge. Every six ducks and six chickens in each groups were challenged with 100 BLD_50_ of H5N6 HPAIV (A/duck/Korea/ES2/2016). Survival rates of ducks and chickens were observed until 12 day post chiallenge.

**Figure 6:**
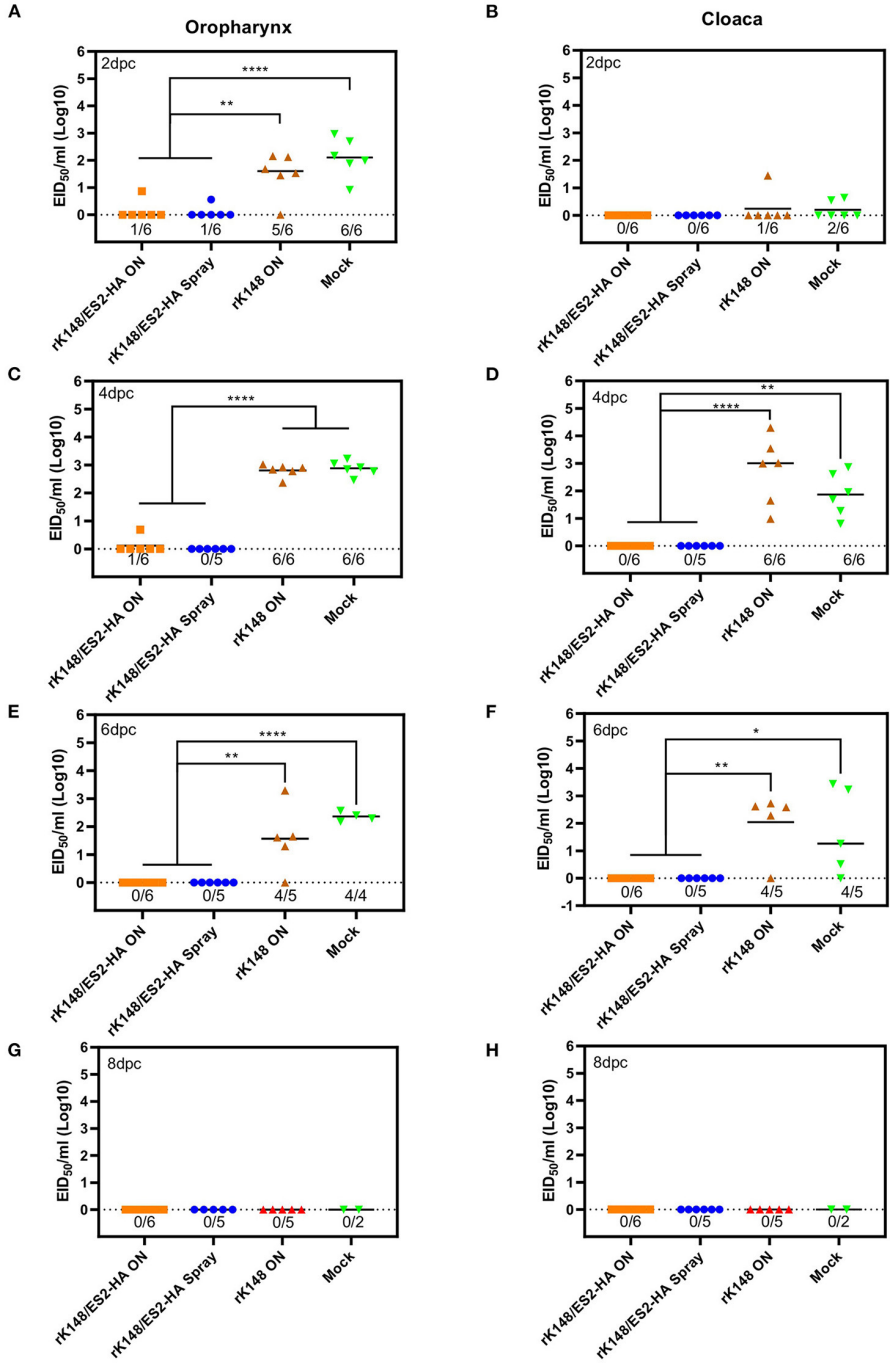
Oropharyngeal and cloacal viral shedding titers of vaccinated ducks and mock vaccinated ducks after H5N6 HPAIV challenge. A total of 24 ducks was divided into four groups and vaccinated with rK148/ES2-HA virus (ON route), rK148/ES2-HA virus (spraying), K148 virus (ON route), and PBS (mock-vaccinated group). Three weeks later, each group received the second vaccination as in the first vaccination. Six weeks after the first vaccination, the ducks were challenged with 100 BLD_50_ of H5N6 HPAIV (A/duck/Korea/ES2/2016). Virus shedding was monitored by quantifying viral RNA using rRT-PCR of oropharyngeal and cloacal swabs collected on 2 **(A,B)**, 4 **(C,D)**, 6 **(E,F)**, and 8 **(G,H)** day post-challenge. (*p < 0.05 and **p < 0.01 using ANOVA with *post-hoc* Tukey's test compared with other groups).

**Figure 7:**
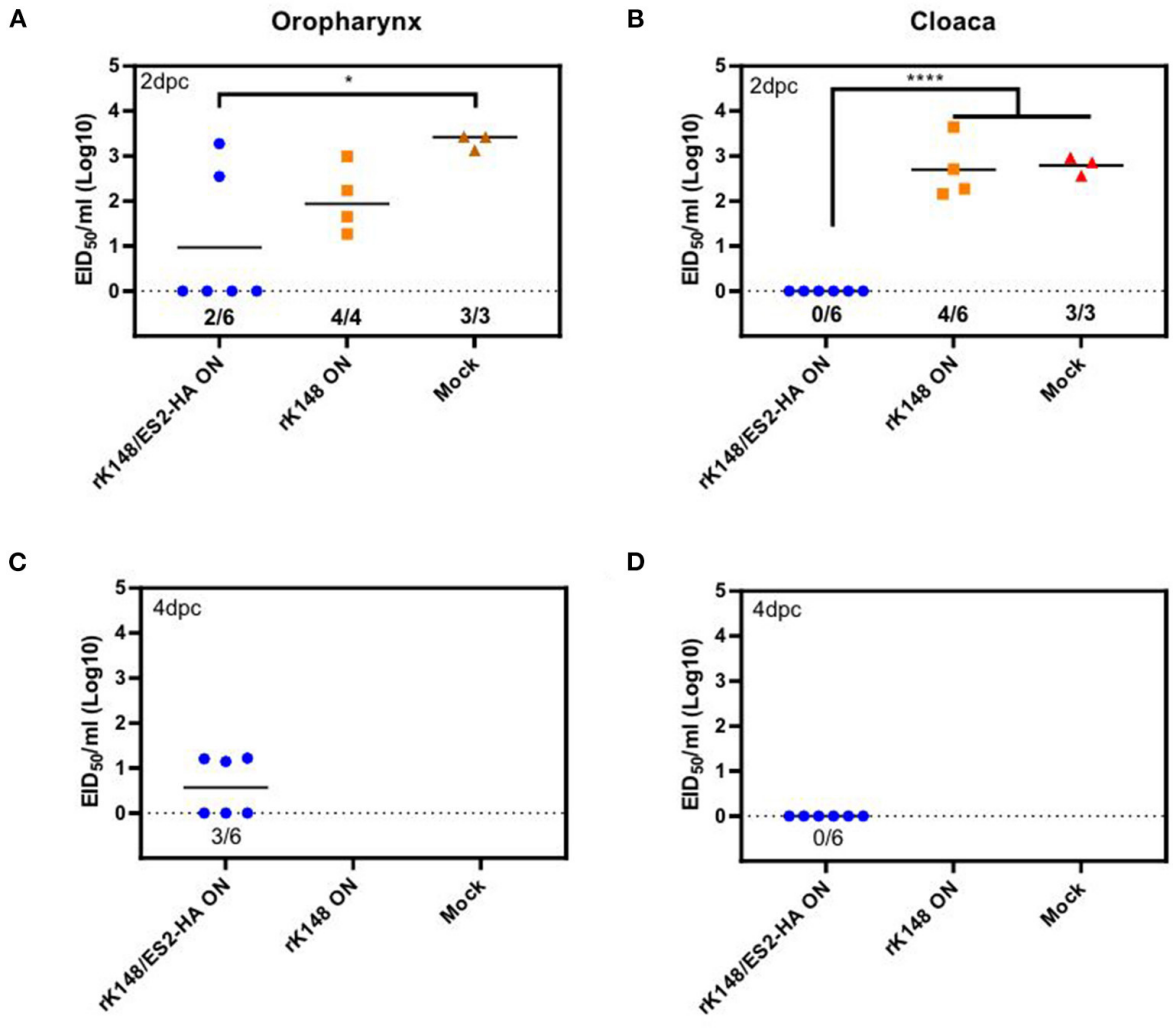
Oropharyngeal and cloacal viral shedding titers of vaccinated and mock-vaccinated chickens after H5N6 HPAIV challenge. A total of 18 chickens were divided into three groups and vaccinated with rK148/ES2-HA virus (ON route), K148 virus (ON route), and PBS (mock-vaccinated group). Chickens were administered booster vaccination and challenged with 100 BLD_50_ of H5N6 HPAIV, and viral shedding was monitored as described in Figure 5. Oropharyngeal and cloacal swabs were collected on 2 **(A,B)**, 4 **(C,D)**, 6 and 8 day post-challenge. (*p < 0.05 and ****p < 0.0001 using ANOVA with *post-hoc* Tukey's test compared with other groups).

To examine the protective efficacy of the rK148/ES2-HA vaccine, we challenged ducks and chickens with 100 BLD_50_ of ES2HPAI virus at 3 weeks after the booster vaccination. In the duck challenge study, the survival rates of the oculonasally vaccinated group (100%) was significantly higher than those of the mock-vaccinated group (33.3%) (*p* = 0.0195; Figure 5A). Interestingly, the rK148 vaccinated group and spray vaccinated group showed the same survival rate of 83%. In the chicken challenge study, all chickens in the rK148-vaccinated and mock-vaccinated groups succumbed on day 3 post-challenge, whereas the rK148/ES2-HA vaccinated group showed a 100% survival rate (Figure 5B). In the viral shedding study of challenged ducks, the mean viral titers in oropharyngeal swab during day 2–6 pc of two vaccinated groups were significantly lower than that of rK148 vaccinated group (10^1.49−2.81^ EID_50_/ml) and mock vaccinated group (10^2.11−2.88^ EID_50_/ml) (*p* < 0.01). Only one out of six ducks in the oculonasally vaccinated or spray-vaccinated group showed virus shedding in the oropharyngeal swab on day 2 pc (10^0.86^ EID_50_/ml and 10^0.56^ EID_50_/ml) with mean viral titers significantly lower than that of the rK148 (10^1.49^ EID_50_/ml) or mock vaccinated group (10^2.11^ EID_50_/ml) (*p* < 0.01 and *p* < 0.0001, respectively; Figure 6A). Cloacal viral sheddings of vaccinated ducks were significantly lower than rK148 vaccinated and mock vaccinated ducks at day 4 and 6 pc (*p* < 0.05). In addition, viral shedding by the rK148-vaccinated and mock-vaccinated groups was observed until day 6 pc, whereas most ducks vaccinated with rK148/ES2-HA did not show viral shedding after day 2 pc (Figure 6). Chickens vaccinated with rK148/ES2-HA *via* the oculonasal route showed lower viral shedding compared to the rK148-vaccinated and mock-vaccinated control groups (Figure 7).

### Clinical Score

Ducks vaccinated with rK148/ES2-HA by oculonasal and spray route did not show any clinical sign. Spray vaccinated ducks have higher clinical score due to one dead duck at day 5 pc. In K148/08 vaccinated group, one duck showed prostration at day 3 pc and died at day 4 pc. Three ducks in same group showed ruffled feather and lethargy starting from day 5 and 7 pc. In positive control group, 4 ducks started to showed lethargy, prostration and torticollis 1–2 days before deaths, and two survived ducks showed lethargy and ruffled feather. Chickens vaccinated with rK148/ES2-HA did not showed any clinical sign after the challenge. All chickens in mock vaccinated group and rK148 vaccinated group showed sudden death at day 2 and 3 pc (Supplementary Figure 1).

## Discussion

In this study, we generated an NDV-vectored vaccine platform using a previously established lentogenic NDV strain (K148/08) from wild waterfowls (31). Thus, the vector vaccine developed benefited by adopting safety, thermostability, immunogenicity, and protective efficacy of K148/08 vaccine against velogenic NDV infection, as evidenced by over 6 years of use in the veterinary market in South Korea. To further utilize the vector vaccine platform for H5 HPAIV, the recombinant NDV vector virus was genetically modified to express the HA coding region of clade 2.3.4.4e ES2 HPAIV flanked by the P and M genes of NDV (Figure 1). We were able to identify the expression of the HA protein of the AIV and HN proteins of NDV in the rescued viruses without significant impairment in the viral growth (Figures 2, 3). In bird experiments, ducks and chickens showed increased HI antibody titers against AIV and NDV and were protected from lethal infection by administering the rK148/ES2-HA vaccine *via* the oculonasal route or spraying (Figures 4, 5). To the best of our knowledge, although there have been several studies on the application of NDV-vectored H5 HPAIV vaccine for use in chickens (15, 36–39), this is the first study to examine the efficacy of the NDV-vector H5 HPAIV vaccine using the spray method in commercial duck species (Pekin duck) most commonly available in Korea.

In the chicken experiment, SPF chickens vaccinated with rK148/ES2-HA elicited HI antibody titer against H5N6 HPAIV and were protected against the H5N6 HPAIV challenge (Figures 4B, 5B). As shown in the HI results of the mock-vaccinated group (Figure 4D), SPF chickens used in this study did not have MDA (Maternally derived antibody) to NDV, but use of recombinant NDV vaccine is limited by the presence of anti-NDV antibodies (29). MDA against NDV is prevalent in most commercial chickens due to routine vaccination against NDV (40, 41). In 2013; Steglich et al. (37) tailored NDV-vectored HPAIV vaccine by substituting the F and HN proteins of NDV to corresponding proteins of avian paramyxovirus type 8, which provided full protection against lethal HPAIV infection in chickens that had MDA against NDV before the vaccination. Likewise, further modification of rK148/ES2-HA can enable the use of vaccine in chickens with MDAs.

In the duck experiment, ducks immunized with rK148/ES2-HA *via* the oculonasal route elicited HI antibody titers against H5N6 HPAIV at 2 weeks after vaccination, whereas the ducks immunized *via* spraying developed significant HI titers only after booster vaccination (Figure 4A). We assumed that while the oculonasal inoculation delivered a constant amount of the vaccine virus, some loss of the vaccine virus was inevitable when delivered *via* the spray method. Although the spray vaccinated group showed late onset of the immunity when compared to the oculonasally vaccinated group (Figure 4), the spray method has advantage in that it can be mass-applicated without labor-intensive administration methods. This advantage will enable the fast vaccine administration to form herd immunity against HPAIV in the field situation. Unlike chickens, low level of HI antibody titer against NDV was observed in mock-vaccinated ducks and in ducks that were not vaccinated (Figure 4C). However, this HI antibody titer was neglectable as HI antibody titers were lower than 4log_2_ and no impairment was observed in protective efficacy of rK148/ES2-HA vaccine. It was also observed in another study in mule ducks that vaccination with rNDV-H5 in the presence of NDV MDA induced lower NDV-specific serum antibody responses than in ducks without MDA, and the H5-specific serum antibody response was higher in ducks with NDV MDA (30). The effect of NDV MDA on rK148/ES2-HA vaccination in ducks remains to be determined. It is noteworthy that the viral shedding in ducks and chickens were significantly lower than that of the rK148 vaccinated group and the mock vaccinated group (Figures 6, 7). Although virus replication was analyzed only through swab samples, as in the results of several studies using recombinant live vaccines, no or low viral shedding in OP and CL route as primary viral replicating tissue suggests limited possibility of virus spread to internal organs (42, 43). In this study 10^7^EID_50_ of rK148/ES2-HA was administrated to birds, which is a high dose considering most vaccine studies based on NDV vector used lower than 10^7^EID_50_ for vaccination in poultry. Additional dose-sparing studies should be conducted to find a dose that can satisfy both the protective potential and economic feasibility of the vaccine. In summary, depending on the delivery method, optimal dose, and time of administration, the recombinant NDV vaccine could protect against HPAIV in ducks.

Interestingly, ducks vaccinated with rK148 showed a higher survival rate (86.3%) than the mock-vaccinated group (Figure 5A). However, the viral titers in swab samples were as high as those in mock-vaccinated ducks. It seems that rK148 triggered local and nonspecific cellular immune responses in the respiratory tract and alleviated the pathogenic effect of H5N6 HPAIV but could not stop viral replication. By contrast, chickens vaccinated with rK148 showed 100% mortality, as chickens were more susceptible to infection of the virus. In addition to the rK148-vaccinated group and mock-vaccinated groups, ducks, and chickens vaccinated with rK148/ES2-HA showed high survival rates and low viral shedding after the viral challenge.

Taken together, timely and proper administration of the rK148/ES2-HA vaccine will help protect ducks and chickens from lethal HPAIV infection and reduce the risk of virus transmission to other poultry species in situations of H5 HPAIV outbreaks.

## Data Availability Statement

The original contributions presented in the study are included in the article/Supplementary Materials, further inquiries can be directed to the corresponding author/s.

## Ethics Statement

The animal study was reviewed and approved by Institutional Animal Care and Use Committee of Konkuk University.

## Author Contributions

JL and C-sS designed the study. JL, D-hK, and J-hJ conducted animal experiments. JL, D-hK, and JN generated plasmids for the generation of the recombinant virus. JL analyzed the data and drafted the manuscript. C-sS, J-bL, S-YP, I-sC, and S-WL supervised the experiments. C-sS, SY, and JN revised the manuscript. All authors read and approved the submission of this manuscript.

## Funding

This work was supported by the Korea Institute of Planning and Evaluation for Technology in Food, Agriculture, Forestry (IPET) through the Animal Disease Management Technology Development Program, funded by the Ministry of Agriculture, Food and Rural Affairs (MAFRA) (grant number: 318032).

## Conflict of Interest

JN and C-sS were employed by company KCAV Co., Ltd. The remaining authors declare that the research was conducted in the absence of any commercial or financial relationships that could be construed as a potential conflict of interest.

## Publisher's Note

All claims expressed in this article are solely those of the authors and do not necessarily represent those of their affiliated organizations, or those of the publisher, the editors and the reviewers. Any product that may be evaluated in this article, or claim that may be made by its manufacturer, is not guaranteed or endorsed by the publisher.
