# Supplementary material for: Live Recombinant NDV-Vectored H5 Vaccine Protects Chickens and Domestic Ducks From Lethal Infection of the Highly Pathogenic H5N6 Avian Influenza Virus
Source: Front Vet Sci. 2022 Feb 3;8:773715. doi: 10.3389/fvets.2021.773715 (PMC8850738; doi:10.3389/fvets.2021.773715)
Supplement: Supplementary file 1 [file Data_Sheet_1.docx]

Supplementary Material

**Supplementary Table 1. Sequences of the primers used to amplify the six fragments of K148 cDNA.**

| **Primer** | **Primer sequences** | **Primer name** |
| --- | --- | --- |
| 1^a^ | 5′-ACCAAACAGAGAATCTGTAAGG-3′ | F1_F |
| 2^a^ | 5′-TCAGTACCCCCAGTCGG-3′ | F1_R |
| 3^a^ | 5′-GTGACCAGATGAGCTTTGC-3′ | F2_F |
| 4^a^ | 5′-TTAGCCATTCAGTGCAAGG-3′ | F2_R |
| 5^a^ | 5′-GTGCATTGATCATGTCACG-3′ | F3_F |
| 6^a^ | 5′-GTCAGTTTGACATTGACCTTG-3′ | F3_R |
| 7^a^ | 5′-GATAGAAGAACTTGACACCTC-3′ | F4_F |
| 8^a^ | 5′-GAGCCATGCAAACTTGGCTG-3′ | F4_R |
| 9^a^ | 5′-ATGGCGRGCTCCGGTCC-3′ | F5_F |
| 10^a^ | 5′-AACAGCATTGCATGCATGC-3′ | F5_R |
| 11^a^ | 5′-GACTAACCTTCAATACTCAAG-3′ | F6_F |
| 12^a^ | 5′-ACCAAACAAAGATTTGGTGAATG-3′ | F6_R |
| 13^b^ | 5′-ATGTCTTCTGTATTCGATGAG-3′ | NP_F |
| 14^b^ | 5′-TCAGTACCCCCAGTCGG-3′ | NP_R |
| 15^b^ | 5′-ATGGCCACCTTYACAGATG-3′ | P_F |
| 16^b^ | 5′-TTAGCCATTCAGTGCAAGG-3′ | P_R |
| 17^b^ | 5′-ATGGCGRGCTCCGGTCC-3′ | L_F |
| 18^b^ | 5′-TTAAGAGTCACAGTTACTRTAATATC-3′ | L_R |

^a^Primers 1–12 were used to amplify six cDNA genome segments of the NDV K148/08 strain.

^b^Primers 13–18 were used to amplify cDNA of the RNP complex genes (NP, P, and L genes).

**Supplementary Table 2. Sequences of the primers used to amplify the cDNA fragments for in-fusion PCR**

| **Primer** | **Primer sequence^d^** | **Primer name** |
| --- | --- | --- |
| 1^a^ | 5′-ACCAAACAGAGAATCTGTAAGGTACG-3′ | F1-F |
| 2^a^ | 5′-gatttggtgaatgacCTCTCATCAAATCCAAAAATTG-3′ | F1-up-trailer |
| 3^a^ | 5′-TGGATTTGATGAGAGCGGTGGCAAATAGC-3′ | F2-F |
| 4^a^ | 5′-gatttggtgaatgacTTAGCCATTCAGTGCAAGGC-3′ | F2-up-trailer |
| 5^a^ | 5′-TGTCACGCCCTATGCATCCGAGCTCC-3′ | F3-F |
| 6^a^ | 5′-gatttggtgaatgacGAGATATCGAGATTGCCTGTC-3′ | F3-up-trailer |
| 7^a^ | 5′-CAATCTCGATATCTCGACTGAGCTTGG-3′ | F4-F |
| 8^a^ | 5′-gatttggtgaatgacAGCCGATTCAAGTATTTTCTTCC-3′ | F4-up-trailer |
| 9^a^ | 5′-ATACTTGAATCGGCTTCTCCTGACAC-3′ | F5-F |
| 10^a^ | 5′-gatttggtgaatgacCTTCCTCCTACCTACGGAGCTTG-3′ | F5-up-trailer |
| 11^a^ | 5′-GTAGGTAGGAGGAAGCAGATTCAGG-3′ | F6-F |
| 12^a^ | 5′-gatttggtgaatgacAGAACTACACTCAAGAACAATTAC-3′ | F6-up-trailer |
| 13^b^ | 5′-GATTCTCTGTTTGGTccctatagtgagtcgtattagc-3′ | F1-up |
| 14^b^ | 5′-CTCTCATCAAATCCAaaaattgggtctc-3′ | F2-up |
| 15^b^ | 5′-GCATAGGGCGTGACAtgatcaatgcac-3′ | F3-up |
| 16^b^ | 5′-GAGATATCGAGATTGcctgtcacgattac-3′ | F4-up |
| 17^b^ | 5′-AGCCGATTCAAGTATtttcttccattgtcg-3′ | F5-up |
| 18^b^ | 5′-CTTCCTCCTACCTACggagcttgcttc-3′ | F6-up |
| 19^b^ | 5′-gtcattcaccaaatctttgtttg-3′ | Trailer-down |
| 20^c^ | TTAGCCATTCAGTGCAAGGCGC | Insertion-up |
| 21^c^ | TCACCACTGCAGCTCGCAG | Insertion-down |
| 22^c^ | 5′-cactgaatggctaaTCACCACTGCAGCTC-3′ | UTR-HA_F |
| 23^c^ | 5′-gagctgcagtggtgaTAATTGCAATTTGAG-3′ | UTR-HA_R |

^a^Primers 1–12 were used to assemble cDNA of the NDV K148/08 strain into the pBlueScript vector.

^b^Primers 13–19 were used to linearize the vector backbone.

^c^Primers 20–23 were used to insert the UTR and HA genome into the pK148 vector backbone.

^d^Nucleotides shown in lower case represent homology sequences with a vector backbone.
